# Comparison of safety and effectiveness of medical adhesive and metal spring coil in preoperative localization of peripheral pulmonary nodules

**DOI:** 10.3389/fmed.2024.1506254

**Published:** 2025-01-13

**Authors:** Yifei Wang, Zhenhua Yue, Xiaoqian Shi, Guozhan Xia, Linlin Qin, Qi Sun, Yiling Huang, Rong Chen, Xuewei Zhao, Mingdong Wang

**Affiliations:** ^1^Department of Thoracic Surgery, Shanghai Fourth People’s Hospital, School of Medicine, Tongji University, Shanghai, China; ^2^Department of Pulmonary and Critical Care Medicine, Shanghai Fourth People’s Hospital, School of Medicine, Tongji University, Shanghai, China; ^3^Department of Nursing, Shanghai Fourth People’s Hospital, School of Medicine, Tongji University, Shanghai, China

**Keywords:** medical adhesive, metal spring coil, localization, pulmonary nodules, video-assisted thoracoscopic surgery (VATS)

## Abstract

**Background:**

Accurate preoperative positioning is the key to the success of thoracoscopic surgery for small pulmonary nodules. There are many methods for locating pulmonary nodules in clinical practice, but there are currently few research reports on the value of medical adhesive localization.

**Objective:**

To compare the clinical value of two positioning methods, medical adhesive and metal spring coil, in the preoperative application of VATS through retrospective analysis.

**Methods:**

A total of 288 patients who underwent thoracoscopic surgery in our hospital from January 2021 to June 2024 due to the discovery of solitary pulmonary nodules during chest CT examination were included in this study. Preoperative patients were randomly divided into two groups, with 205 patients undergoing preoperative medical adhesive positioning (Group A) and 83 patients undergoing metal spring coil positioning (Group B). After the positioning was completed, record the positioning time of each group of patients and the immediate pain score 15 min after the positioning was completed, the complications located in each group of patients, and whether there was positioning failure or not.

**Results:**

The localization success rate of the medicine adhesive positioning group [99.5% (204/205)] was higher than that of the metal spring coil positioning group [91.6% (76/83)] (*P* = 0.001). The positioning time of the medical adhesive positioning group was 12.00 (10.00, 14.00) min, which was shorter than the 13.00 (11.00, 16.00) min of the micro coil group (*P* = 0.001). The immediate pain score (2.32 ± 0.79) of the medical adhesive positioning group 15 min after positioning was significantly lower than that of the metal spring coil positioning group (3.97 ± 0.54) (*P* < 0.001). The incidence of complications such as pneumothorax [15.7% (13/83) vs 5.4% (11/205), *P* = 0.004], pulmonary hemorrhage/hemoptysis [20.5% (17/83) vs 4.9 (10/205), *P* < 0.001] was significantly higher in the metal coil positioning group than in the medical adhesive positioning group.

**Conclusion:**

Preoperative medical adhesive positioning for pulmonary nodules is safe, reliable, and effective. Compared with metal spring coil positioning, it has shorter positioning time, milder pain after positioning, lower incidence of positioning related complications, and more flexible arrangement of surgical timing after positioning. It has high clinical application value.

## 1 Introduction

With the gradual popularization of low-dose thin-layer CT in early lung cancer screening, the detection rate of pulmonary nodules is gradually increasing (1). For small pulmonary nodules, if early lung cancer is highly suspected, video-assisted thoracoscopic surgery (VATS) should be the first choice for resection to clarify the diagnosis and achieve treatment goals (2, 3). However, for small pulmonary nodules with small diameter, insufficient solid components, or not close to the pleura, it is often difficult to accurately locate them during surgery, which may lead to prolonged surgery time, conversion to thoracotomy, and even surgical failure. Especially for smaller pure ground glass nodules, intraoperative localization is more difficult due to the lack of solid components. Therefore, accurate preoperative positioning is the key to surgical success (4, 5). Whether it can be accurately positioned is related to the success rate of surgery and patient efficacy. To ensure surgical effectiveness and patient safety, and to enable clinical physicians to quickly and accurately locate lung nodules during thoracoscopic surgery, it is particularly important to choose the correct and reasonable positioning method before surgery. Adopting a technique that can accurately locate pulmonary nodules before surgery can avoid unnecessary removal of normal lung tissue in patients with pulmonary nodules, which will be beneficial for their recovery (6).

At present, there are many methods for locating pulmonary nodules in clinical practice (7), such as traditional hook-wire positioning, metal spring coil positioning, as well as emerging medical adhesive positioning, metal anchoring needle positioning, and so on. Although there have been research reports on preoperative localization of pulmonary nodules, it is not yet clear which method is more advantageous. Previous study have shown that CT-guided percutaneous localization with medical adhesive can label small pGGNs and mGGNs prior to VATS, with high success and low complication rates (8). There is also study indicating that, for pulmonary nodules that are difficult to locate in VATS, CT guided coil positioning can help doctors accurately locate these nodules and make it easier and faster to remove them (9). However, there are currently few research reports on the positioning value of medical adhesives. Our study aims to compare the clinical value of two positioning methods, medical adhesive and metal spring coil, in the preoperative application of VATS through retrospective analysis. Hook-wire positioning was excluded from our study due to its sharp tip and severe post positioning pain, and metal anchoring needle positioning were also excluded from our study because their use was limited by that the positioning needle must be resected during surgery after positioning.

## 2 Materials and methods

### 2.1 Clinical data and grouping

A total of 288 patients who underwent thoracoscopic surgery in our hospital from January 2021 to June 2024 due to the discovery of solitary pulmonary nodules during chest CT examination were included in this study. Preoperative patients were randomly divided into two groups, with 205 patients undergoing preoperative medical adhesive positioning (Group A) and 83 patients undergoing metal spring coil positioning (Group B). There were 66 males and 139 females in the adhesive localization group, aged 21–82 years, with an average of 57.43 ± 12.70 years; the target nodules were located in the upper lobe of the right lung in 52 cases, the middle lobe of the right lung in 7 cases, the lower lobe of the right lung in 44 cases, the upper lobe of the left lung in 57 cases, and the lower lobe of the left lung in 45 cases. In the metal spring coil positioning group, there were 29 males and 54 females with ages ranging from 27 to 81 years, with an average of 54.77 ± 12.19 years; the target nodules were located in the upper lobe of the right lung in 20 cases, the middle lobe of the right lung in 7 cases, the lower lobe of the right lung in 24 cases, the upper lobe of the left lung in 17 cases, and the lower lobe of the left lung in 15 cases. All patients had no perioperative deaths.

### 2.2 Inclusion and exclusion criteria

Inclusion criteria were the following: (1) conventional thoracoscopic surgery; (2) preoperative CT scan confirmed the presence of isolated peripheral pulmonary nodules, and malignancy cannot be ruled out; (3) The distance between the pulmonary nodule and the pleural surface is greater than 5 mm, and precise positioning may not be possible through touch during surgery. Exclusion criteria were: (1) those who underwent thoracic surgery for more than one time on the same side; (2) patients with obvious abnormalities in blood biochemical examination within 7 days before surgery (e.g., liver function damage, renal function damage, et al.); (3) severe cardiac insufficiency (NYHA grade III IV), poor blood pressure control in hypertensive patients or poor blood glucose control in diabetic patients; (4) those with adhesion in the pleural cavity; (5) patients with pulmonary bullae, chronic obstructive pulmonary disease, and other diseases are prone to pneumothorax due to puncture positioning; (6) those whom researchers evaluated as not suitable for inclusion, i.e., patients with poor compliance, hearing impairment or communication impairment, or lost to follow-up.

### 2.3 Instruments and equipment

The CT equipment used is Lianying high-resolution 64 slice spiral CT scanner. The medical adhesive used for positioning were Kangpaite medical adhesive (0.5 ml/tube) and the matched puncture needle (specification: 21G, length: 80 mm) produced by Beijing Kangpaite Medical Equipment Co., Ltd. The metal spring coil positioning used for positioning was German SOMATEX disposable breast positioning wire and its guide pin (length: 100 mm, specification: 20G, diameter: 0.95 mm) produced by Shanghai Songke Medical Equipment Co., Ltd.

### 2.4 Methods

All patients underwent pulmonary nodule localization within 8 h prior to VATS surgery, which was performed by the same respiratory physician with over 15 years of work experience. Firstly, based on preoperative CT examination to clarify the spatial relationship of pulmonary nodules, the optimal puncture path is planned and selected, and the optimal puncture position (left/right lateral position, supine position, prone position) is determined. Then, a local thin-layer CT scan (layer thickness: 1.00 mm) is performed to determine the puncture point, angle, and depth. Disinfect and drape the area of 15 square centimeters near the puncture site, and use 5 ml of 2% lidocaine to infiltrate and anesthetize the chest wall and pleura at the puncture site. Instruct the patient to breathe as calmly as possible.

Medical adhesive positioning group (Group A): Insert the needle according to the predetermined angle and depth, instruct the patient to hold their breath after inhalation, and insert the puncture needle based on the measured optimal depth. Then perform a local CT thin-layer scan to observe the position relationship between the puncture needle and the lesion. After the puncture needle reaches 5–10 mm around the lesion and 10–25 mm deep into the lungs, remove the needle core, and after confirming that there is no blood return, quickly inject 0.15 ml of medical gel that has been extracted into the lungs through the trocar, so that the medical gel was injected into the lung tissue and quickly solidifies to form a hard knot. Then, remove the trocar to complete the positioning. Cover the wound with a band aid or medical dressing. After the positioning was completed, according to the surgical schedule, the patient was sent back to the ward to wait for the surgery.

Metal spring coil positioning group (Group B): Insert the needle according to the predetermined angle and depth, instruct the patient to hold their breath after inhalation, and insert the puncture needle based on the measured optimal depth. Then perform a local CT thin-layer scan to observe the position relationship between the puncture needle and the lesion. After the puncture needle reaches 5–10 mm around the lesion and 10–25 mm deep into the lungs, gradually release the guide wire until it cannot be pushed, and be careful to avoid the guide needle moving backwards. Slowly remove the guide needle, taking care to avoid the guide needle moving backwards. Apply and fix locally to prevent the guide wire from shifting.

**FIGURE 1:**
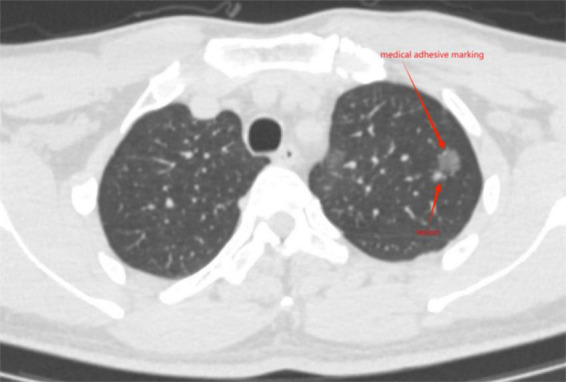
Medical adhesive marking located near the lesion.

**FIGURE 2:**
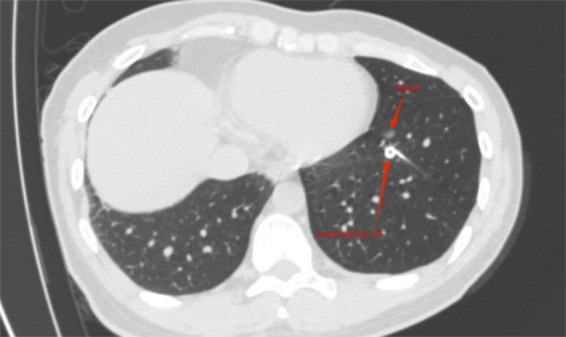
Metal spring coil located near the lesion.

Immediately perform another local thin-layer CT scan after localization to confirm the location of medical adhesive nodules (Figure 1) or metal spring coils (Figure 2), as well as the presence of complications such as pneumothorax, pulmonary bleeding, or hemoptysis, and promptly report the localization effect to the thoracic surgeon. Instruct the patient to sit quietly and observe for 15 min, avoid vigorous exercise, and return to the ward to wait for surgery after no abnormalities are found.

Under general anesthesia with double lumen endotracheal intubation, take a lateral position with the affected side facing upwards. Routine disinfection and cloth laying were performed, and all patients underwent thoracoscopic surgery using the two hole method. A 1.0 cm incision was made at the midline of the 7th rib axilla, and thoracoscopy was inserted; Make a 3 cm operating hole in the fourth intercostal space of the axillary line to explore and determine the depth of medical adhesive or metal spring coil positioning and the location of the lesion. According to the positioning, perform wedge resection or segmentectomy of the lung, and simultaneously remove the medical adhesive or metal spring coil used for positioning during the operation.

### 2.5 Record observation indicators

(1) After the positioning was completed, promptly record the positioning time of each group of patients and the immediate pain score 15 min after the positioning is completed. The positioning time started from the CT scan that determines the puncture path and ended after the positioning was completed, accurate to min. After the positioning was completed, the nurse assisting in the positioning was responsible for calculating the positioning time and recording it. The immediate pain score 15 min after positioning was recorded using a VAS pain scale combined with the digital score scale (accurate to 0.1).

(2) Record the complications located in each group of patients (such as pneumothorax, pulmonary hemorrhage or hemoptysis, pleural reaction, etc.), and analyze the influencing factors of the complications.

(3) Intraoperative records of VATS: Surgical date, surgical method (wedge resection or segmentectomy), presence or absence of intrathoracic hemorrhage; is there any medical adhesive detachment/coil displacement or dislodgement that causes the inability to locate the target nodule during surgery (positioning failure).

(4) Postoperative records of VATS: Pathological results and pathological size of every localized nodules.

### 2.6 Statistical analysis

IBM SPSS Statistics 21.0 software was used for statistical analysis. Firstly, use the Kolmogorov-Smirnov method to verify whether the metric data conforms to a normal distribution. The measurement data which has undergone normality testing conformed to normal distribution were represented by mean ± standard deviation. Independent sample t-test was used for inter group comparison. Data that do not follow normal distribution were represented by the median (quartiles), and comparisons between groups were conducted using the Mann-Whitney U test. The categorical count data were represented by the number of cases (%), and the comparison between groups was performed using the χ^2^ test or Fisher’s exact probability test. The inspection level was all *P* < 0.05, indicating that the difference was statistically significant.

### 2.7 Ethical approval

Ethics committee of Shanghai Fourth People’s Hospital and Internal Review Board of Shanghai Fourth People’s Hospital have approved this study. Each patient signed an informed consent form before positioning. Declaration of Helsinki and International Ethical Guidelines for Health-related Research Involving Humans are followed. The patients have provided consent for participating the study and publication of the data on any journal.

## 3 Results

### 3.1 Comparison of general patient information

The comparison of general clinical data between two groups of patients is shown in Table 1. The difference in gender composition, location of nodules, surgical approach, and proportion of postoperative pathological types between the two groups of patients was not statistically significant (*P* > 0.05) according to the results of the χ^2^ test. In addition, independent sample t-test results showed no statistically significant differences in age and pathological size of nodules between the two groups of patients (*P* > 0.05).

**TABLE 1:** Demographic and clinical characteristics of the 288 patients in the study.

	Group A (*n* = 205)	Group B (*n* = 83)	χ^2^/*t*	*P*
Age, mean ± SD, y	57.43 ± 12.70	54.77 ± 12.19	1.631	0.104
**Gender, No. (%)**				
Male	66(32.2)	29(34.9)	0.201	0.654
Female	139(67.8)	54(65.1)		
**Location of nodules, No. (%)**				
Right upper lobe	52(25.4)	20(24.1)	6.149	0.188
Right middle lobe	7(3.4)	7(8.4)		
Right lower lobe	44(21.5)	24(28.9)		
Left upper lobe	57(27.8)	17(20.5)		
Left lower lobe	45(21.9)	15(18.1)		
Pathological size of nodules, mean ± SD, mm	5.01 ± 2.57	5.04 ± 2.13	0.083	0.934
**Surgical approach, No. (%)**				
Wedge	200(97.6)	83(100.0)	0.879	0.349
Segmentectomy	5(2.4)	0(0.0)		
**Postoperation pathology, No. (%)**				
Benign tumor	56(27.3)	29(35.0)	1.650	0.199
**Malignant tumor**				
AAH	21(10.3)	4(4.8)	2.209	0.530
AIS	80(39.0)	29(35.0)		
MIA	39(19.0)	16(19.2)		
IA	9(4.4)	5(6.0)		

AAH, atypical adenomatous hyperplasia; AIS, adenocarcinoma *in situ*; MIA, minimally invasive adenocarcinoma; IA, invasive adenocarcinoma.

### 3.2 Comparison of positioning time and immediate pain scores 15 min after positioning between two groups of patients

The comparison of the positioning time and immediate pain scores 15 min after positioning between the two groups of patients is shown in Table 2. The Mann-Whitney U test results showed that the positioning time of Group A was significantly lower than that of Group B, and the difference was statistically significant (*P* < 0.05). The independent sample t-test results showed that the immediate pain scores 15 min after positioning of Group A was significantly lower than that of Group B, and the difference was statistically significant (*P* < 0.05).

**TABLE 2:** Comparison of positioning time and immediate pain scores 15 min after positioning between two groups of patients.

	Group A	Group B	*Z/t*	*P*
Number of cases	205	83	–	–
Positioning time [min, M(P_25_,P_75_)]	12.00 (10.00, 14.00)	13.00 (11.00, 16.00)	−3.454	0.001
Immediate pain scores 15 min after positioning ($\bar{x}\pm S$)	2.32 ± 0.79	3.97 ± 0.54	20.512	<0.001

### 3.3 Comparison of location related complications and positioning failure rate between two groups of patients

The comparison of location related complications between the two groups of patients is shown in Table 3. Both groups of patients did not experience any intrathoracic bleeding. The results of the χ^2^ test showed that the proportion of pneumothorax, intrapulmonary bleeding/hemoptysis, medical adhesive detachment/coil displacement or dislodgement (positioning failure) in Group B was significantly higher than that in Group A, and the difference was statistically significant (*P* < 0.05). The Fisher’s exact probability test results showed that there was no statistically significant difference in the proportion of pleural reactions between the two groups of patients (*P* > 0.05).

**TABLE 3:** Comparison of location related complications and positioning failure rate between two groups of patients [*n* (%)].

Complications	Group A (*n* = 205)	Group B (*n* = 83)	χ^2^	*P*
Pneumothorax	11 (5.4)	13 (15.7)	8.200	0.004
Intrapulmonary bleeding/Hemoptysis	10 (4.9)	17 (20.5)	16.931	<0.001
Intrathoracic bleeding	0 (0.0)	0 (0.0)	–	–
Pleural reaction	0 (0.0)	1 (1.2)	–	0.288*
Medical adhesive detachment/Coil displacement or dislodgement (positioning failure)	1 (0.5)	7 (8.4)	11.027	0.001

*Fisher’s exact probability test is used.

## 4 Discussion

The screening of lung cancer has led to an increase in the detection rate of solitary pulmonary nodules, and more than half of the postoperative pathological confirmation of solitary pulmonary nodules is malignant tumors. The diagnosis and treatment of pulmonary nodules have become an increasingly serious clinical problem (10, 11). The presence of nodules puts immense psychological pressure on patients, and long-term follow-up may also lead to disease progression. Therefore, surgeons often advocate taking active diagnostic and treatment measures, clarifying benign and malignant conditions, and implementing standardized treatment to reduce the mortality rate associated with lung cancer (12). Minimally invasive thoracoscopic surgery has become the preferred method for diagnosing and treating uncertain isolated pulmonary nodules due to its small trauma and fast postoperative recovery (13), but the detection effect of pulmonary nodules in thoracoscopic surgery is not ideal. Previous studies (14) have shown that up to 54% of lung nodules cannot be observed in VATS or detected by palpation, and for pulmonary ground glass nodules with a diameter <10 mm and located more than 5 mm above the pleural surface, VATS is difficult to locate. Preoperative localization of pulmonary nodules is particularly crucial as it can improve surgical efficiency and accuracy.

Since Plunkett et al. (15) first reported the high efficiency of using Hook wire to locate pulmonary nodules before surgery in 1992, multiple different preoperative localization methods including hook-wire, coil, staining material, and iodine oil have been applied, each with its own unique advantages (16, 17). Preoperative localization results in a higher success rate, shorter surgical time, and faster patient recovery for thoracoscopic lung nodule surgery (18).

Various positioning methods have their own advantages and disadvantages, and the positioning methods chosen by each unit are not the same based on specific technical conditions, clinical experience, and the actual situation of the patient.

The localization success rate of the medicine adhesive positioning group in this study was 99.5% (204/205), and the localization success rate of the metal spring coil positioning group was 91.6% (76/83). It can be seen that both localization techniques can effectively complete preoperative localization, but the localization success rate of the medical adhesive positioning group was significantly higher than that of the metal spring coil positioning group, and the difference was statistically significant (*P* = 0.001). According to literature (19), the dislocation rate of spring coil positioning is reported to be 0–6.7%. The incidence of coil dislocation in this study was slightly higher than the result, at 8.4%. This may be because with the patients’ respiratory movement and the passage of time, some patients may experience displacement of the spring coil, leading to dislocation.

The positioning time of the medical adhesive positioning group was 12.00 (10.00, 14.00) min, which was shorter than the 13.00 (11.00, 16.00) min of the micro coil group, and the difference was statistically significant (*P* = 0.001). This indicates that the process of medical adhesive positioning is more convenient and efficient.

The immediate pain score (2.32 ± 0.79) of the medical adhesive positioning group 15 min after positioning was significantly lower than that of the metal spring coil positioning group (3.97 ± 0.54), and the difference was statistically significant (*P* < 0.001). After the metal spring coil positioning, the head end of the coil was retained in the lung tissue, but the metal tail end needed to penetrate the chest wall and was retained outside the chest wall. Some patients even dared not breathe due to the intense pain; while during the medical adhesive positioning, the puncture needle was promptly removed and there was no residue on the chest wall, which greatly reduced the patients’ pain and made the patients’ movement more convenient and free after positioning.

In terms of complications related to localization, neither group of patients experienced thoracic bleeding, and the perioperative mortality rate was 0, indicating that both medical adhesive and metal spring coil are safe localization methods. There was no statistically significant difference in the incidence of pleural reactions between the two groups of patients in this study (P > 0.05), but the incidence of comorbidities such as pneumothorax [15.7% (13/83) vs 5.4% (11/205), *P* = 0.004], pulmonary hemorrhage/hemoptysis [20.5% (17/83) vs 4.9 (10/205), *P* < 0.001] was significantly higher in the metal coil positioning group than in the medical adhesive positioning group, and the difference was statistically significant.

Medical adhesive is considered an ideal adhesive material and has been widely used in the treatment of bleeding, fistulas, gastrointestinal diseases, and other aspects (20). The full name of medical adhesive is alpha cyanoacrylate rapid medical adhesive, which is made by adding methyl methacrylate to alpha cyanoacrylate octyl ester as the main adhesive. When medical adhesives encounter trace amounts of anionic substances (such as blood, body fluids, tissue fluids, or organic amines in the human body), they quickly polymerize at room temperature, solidify into a film, and tightly embed with the surface of the tissue in contact. They have the function of blocking blood vessel ends and promoting blood vessel contraction, which also has a positive effect on promoting blood coagulation and avoiding pneumothorax. Medical adhesive is non-toxic and harmless, with good biological safety. Medical adhesive localization is achieved by injecting it into the lungs through percutaneous puncture guided by CT, and utilizing its mechanism of action to form a hard lump near the lesion, thus playing a role in localization.

The advantages of medical adhesive positioning are as follows: (1) Simple operation: When using medical adhesive for pulmonary nodule positioning, the operation is not complicated and easy to master; (2) Accurate positioning: Medical adhesive can quickly solidify in the body, and after solidification and hardening, it can ensure accurate positioning. Moreover, medical adhesive solidifies into a hard block in the puncture site tissue, without spreading on the pleural surface and lung parenchyma; (3) Flexible surgical timing: Medical adhesive condenses into hard particles within the tissue at the puncture site, which will not spread on the pleural surface or lung parenchyma. Therefore, positioning can be performed 72 h before surgery, avoiding the defect of hook-wire positioning requiring timely surgery within 3 h. Especially for multiple patients who need preoperative positioning, there is greater room for choice in the selection of positioning dates, and this advantage is even more obvious; (4) Clear tactile sensation: Medical adhesive can quickly solidify in the body, and after solidification and hardening, it can ensure accurate positioning; (5) Reduce complications after positioning: After the medical adhesive solidifies, it produces a large adhesive strength, which can block the broken ends of blood vessels, promote blood vessel contraction and coagulation, immediately stop bleeding, and reduce complications such as puncture bleeding and air leakage; (6) Small impact after positioning: The medical adhesive will not be absorbed in a short period of time after curing, allowing patients to have more freedom of position and feel more comfortable without affecting their activities; (7) Degradable: Medical adhesive is biodegradable, and the hardened adhesive does not need to be removed together; (8) Suitable for patients with multiple nodules: When multiple nodules need to be located simultaneously, hook wire and other metal tools can easily cause pneumothorax and mutual interference. Using medical adhesive for positioning is more convenient, and adhesive hardening will not interfere with the positioning of other nodules.

In our clinical application practice, we have summarized several precautions in the positioning process of medical adhesive: (1) For the positioning of pure ground glass pulmonary nodules, it is necessary to maintain a certain positioning distance to avoid medical adhesive covering the nodules and interfering with pathological diagnosis; (2) The speed of medical adhesive injection should be fast, and the needle should be removed immediately after completion. Delaying the needle removal may cause difficulty or lung injury due to the solidification of the adhesive.

This study still has certain limitations: It is a single center retrospective clinical study, and future multi center, larger sample randomized controlled trials are needed for validation. Despite these limitations, our study provides new insights into preoperative localization techniques.

In summary, preoperative medical adhesive positioning for pulmonary nodules is safe, reliable, and effective. Compared with metal spring coil positioning, it has shorter positioning time, milder pain after positioning, lower incidence of positioning related complications, and more flexible arrangement of surgical timing after positioning. It has high clinical application value.

## Data Availability

The raw data supporting the conclusions of this article will be made available by the authors, without undue reservation.
